# Ethnobotany, ethnopharmacology, and phytochemistry of traditional medicinal plants used in the management of symptoms of tuberculosis in East Africa: a systematic review

**DOI:** 10.1186/s41182-020-00256-1

**Published:** 2020-08-14

**Authors:** Samuel Baker Obakiro, Ambrose Kiprop, Isaac Kowino, Elizabeth Kigondu, Mark Peter Odero, Timothy Omara, Lydia Bunalema

**Affiliations:** 1 Department of Pharmacology and Therapeutics, Faculty of Health Sciences, Busitema University, P.O. Box 1460, Mbale, Uganda; 2 Department of Chemistry and Biochemistry, School of Sciences and Aerospace Studies, Moi University, P.O. Box 3900-30100, Eldoret, Kenya; 3 Africa Centre of Excellence II in Phytochemicals, Textiles and Renewable Energy (ACE II PTRE), Moi University, P.O. Box 3900-30100, Eldoret, Kenya; 4 Department of Pure and Applied Chemistry, Faculty of Science, Masinde-Muliro University of Science and Technology, P.O. Box 190-50100, Kakamega, Kenya; 5 Centre of Traditional Medicine and Drug Research, Kenya Medical Research Institute, P.O. Box 54840-00200, Nairobi, Kenya; 6 Department of Quality Control and Quality Assurance, Product Development Directory, AgroWays Uganda Limited, Plot 34-60, Kyabazinga Way, P.O. Box 1924, Jinja, Uganda; 7 Department of Pharmacology and Therapeutics, School of Biomedical Sciences, Makerere University College of Health Sciences, P.O. Box 7062, Kampala, Uganda

**Keywords:** Antimycobacterial, Antitubercular, Medicinal plants, Herbal medicine, Phytochemicals, *Mycobacterium tuberculosis*

## Abstract

**Objective:**

Many studies on the treatment of tuberculosis (TB) using herbal medicines have been undertaken in recent decades in East Africa. The details, however, are highly fragmented. The purpose of this study was to provide a comprehensive overview of the reported medicinal plants used to manage TB symptoms, and to analyze scientific reports on their effectiveness and safety.

**Method:**

A comprehensive literature search was performed in the major electronic databases regarding medicinal plants used in the management of TB in East Africa. A total of 44 reports were retrieved, and data were collected on various aspects of the medicinal plants such as botanical name, family, local names, part(s) used, method of preparation, efficacy, toxicity, and phytochemistry. The data were summarized into percentages and frequencies which were presented as tables and graphs.

**Results:**

A total of 195 species of plants belonging to 68 families and 144 genera were identified. Most encountered species were from Fabaceae (42.6%), Lamiaceae (19.1%), Asteraceae (16.2%), and Euphorbiaceae (14.7%) families. Only 36 medicinal plants (18.5%) have been screened for antimycobacterial activity. Out of these, 31 (86.1%) were reported to be bioactive with minimum inhibitory concentrations ranging from 47 to 12,500 μg/ml. Most tested plant extracts were found to have acceptable acute toxicity profiles with cytotoxic concentrations on normal mammalian cells greater than 200 μg/ml. The most commonly reported phytochemicals were flavonoids, terpenoids, alkaloids, saponins, cardiac glycosides, and phenols. Only *Tetradenia riparia*, *Warburgia ugandensis*, and *Zanthoxylum leprieurii* have further undergone isolation and characterization of the pure bioactive compounds.

**Conclusion:**

East Africa has a rich diversity of medicinal plants that have been reported to be effective in the management of symptoms of TB. More validation studies are required to promote the discovery of antimycobacterial drugs and to provide evidence for standardization of herbal medicine use.

## Background

Tuberculosis (TB) is a chronic infectious bacterial disease caused by *Mycobacterium tuberculosis* (Mtb). It affects mainly the respiratory system but may also affect other organs of the body causing pulmonary and extrapulmonary TB respectively. The World Health Organization (WHO) estimated that a quarter of the world’s population is infected with Mtb and thus at a risk of developing TB [1]. Although TB affects all people, those living with HIV/AIDS are at a higher risk of developing active TB [2]. The burden of TB is still high as it is ranked among the ten diseases of global concern [3]. In 2018, a total of 10 million new cases and 1.49 million deaths due to TB were reported worldwide. In East Africa, 378,000 new cases and 91,000 deaths (24%) occurred. In East Africa, Kenya and Tanzania are still ranked among the 30 countries with a high burden of TB in the world [1].

Treatment of TB remains a challenge due to the emergence of multidrug-resistant Mtb strains and extensively drug-resistant TB cases which poorly respond to the first line antitubercular drugs (rifampicin, isoniazid, pyrazinamide, and ethambutol). These drugs also have side effects and a high potential to interact with antiretroviral drugs resulting in increased toxicity, poor compliance, and treatment failure [4–6]. As a result, many TB patients have resorted to using alternative and complementary medicines with herbal remedies being the most widely used in the management of tuberculosis [7]. Due to limited access to health services and chronic poverty in East Africa, many people not only believe that herbal medicines are efficacious and safe but also affordable, available, and culturally acceptable [8–10]. Thus, there is widespread use of herbal remedies by many people in the East Africa to manage symptoms of TB [7–13]. The WHO also reported that approximately 60% of the world’s population depend on non-conventional therapies for primary health care [14].

The search to discover new effective drugs against Mtb has intensified globally in the last decade as the current therapies become less effective and in an attempt to have a world free of TB by 2035 [1]. With natural products being the leading sources of novel drugs, ethnobotanical surveys and scientific validation studies have been conducted on East African flora in the past decades [7–10]. Several plant species have been documented and some of their extracts, fractions, and isolated pure compounds have been tested for efficacy and safety [15–18]. However, this information is highly fragmented.

Comprehensive data on medicinal plants used in the management of TB is important for the conservation of these species as some of them are either rare or endangered. It also provides more evidence that increases the confidence in the utilization of these herbal remedies for primary health care as well as their regulation by relevant authorities in case of ineffectiveness and toxicity [19, 20]. The analysis and synthesis of the results may also help in identifying existing gaps and challenges in the current research and stimulates future research opportunities. This can lead to identification of novel molecules that can be developed into new antitubercular drugs with better efficacy and safety profiles [21]. This review was therefore undertaken to compile a comprehensive report on the ethnobotany, ethnopharmacology, and phytochemistry of medicinal plants used in management of symptoms of TB in the East African region so as to generate knowledge on the current status and future opportunities for drug discovery against TB.

## Methods

### Reporting and protocol registration

This systematic review was reported according to the Preferred Reporting Items for the Systematic Reviews and Meta-Analyses (PRISMA) guidelines [22]. The protocol used in this study was registered with the International Prospective Register of Systematic Reviews (PROSPERO) and can be accessed at their website (https://www.crd.york.ac.uk/prospero/display_record.php?RecordID=187098) with the registration number CRD42020187098.

### Literature search strategy

Relevant literature pertaining the ethnobotany, phytochemistry, efficacy and safety of medicinal plants utilized in management of symptoms of TB in Uganda, Kenya, Tanzania, Rwanda, Burundi and South Sudan were retrieved from Scopus, Web of Science Core Collection, PubMed, Science Direct and Google Scholar [23–25]. Key search words such as tuberculosis, *mycobacteria*, tuberculosis symptoms, tuberculosis treatment, vegetal, antituberculosis, antitubercular, antimycobacterial, cough, traditional medicine, ethnobotany, alternative medicine, and ethnopharmacology combined with either Uganda, Kenya, Tanzania, Rwanda, Burundi, or South Sudan were used. All publishing years were considered, and reports in the returned results were carefully scrutinized. More searches were carried out at the Google search engine using more general search terms, such as *mycobacteria*, tuberculosis, antituberculosis, antimycobacterial, cough, vegetal species, vegetal extract, traditional medicine, alternative medicine, plants, plant extract, vegetal, herbal, complementary therapy, natural medicine, ethnopharmacology, ethnobotany, herbal medicine, herb, herbs, decoction, infusion, macerate, and concoction combined with either Uganda, Kenya, Tanzania, Rwanda, Burundi, or South Sudan. The searches were done independently by the authors for each country and the outputs were saved where possible on databases and the authors received notifications of any new searches meeting the search criteria from Science Direct, Scopus, and Google scholar.

### Inclusion and exclusion criteria

Only full-text original research articles published in peer-reviewed journals, books, theses, dissertations, patents, and conference papers on plants used in the management of symptoms of TB in Uganda, Kenya, Tanzania, Rwanda, Burundi, and South Sudan written in English and dated until April 2020 were considered.

### Study selection

At first, literature screening of the extracted articles involved examining the titles and abstracts for relevant articles for inclusion. This was conducted independently by 6 authors. Then, the full-text articles were evaluated against the inclusion/exclusion criteria. The article selection process resulted in 44 studies included in this systematic review (Figure S1).

### Data collection

A data collection tool was designed in Microsoft Excel (Microsoft Corporation, USA) to capture data on different aspects of medicinal plant species used in TB management. These included botanical name, plant family, local name(s), part(s) used, growth habit, mode of preparation and administration, method of extraction, efficacy, toxicity and phytochemical screening of crude extracts, isolated pure compounds, and efficacy and toxicity. Careful review of the articles was done, and data were captured using the tool. The collected data were checked for completeness, processed independently for each country by the authors and later analyzed.

### Data analysis

Missing information in some studies (local names and growth habit of the plants), and misspelled botanical names were retrieved from the Google search engine and botanical databases (The Plant List, International Plant Names Index, NCBI taxonomy browser, and Tropicos) respectively.

Descriptive statistical methods were used to analyze the collected data. Results were expressed as ranges, percentages, and frequencies and subsequently presented as tables and charts. The analyses were performed using SPSS statistical software (Version 20, IBM Inc.)

## Results and discussion

### Ethnobotanical studies

With the current antitubercular drugs becoming less effective in the management of multidrug-resistant Mtb strains, medicinal plants can provide the novel molecules for development of new efficacious and safe drugs [26, 27]. From the electronic survey in multidisciplinary databases, 44 reports on medicinal plants used for management of symptoms of TB in East Africa were retrieved. A total of 195 species of plants belonging to 68 families and 144 genera were identified (Table 1). Some of these documented plant species have also been reported in other regions across the world for management of TB. For example, *Psidium guajava*, *Catha edulis*, *Carica papaya*, *Citrus limon*, *Lantana camara*, *Aloe vera*, *Biden pilosa*, *Piliostigma thonningii*, *Tamarindus indica*, *Ficus platyphyla*, and *Vernonia cinereal* in Nigeria, South Africa, Ethiopia, India, and Mexico [60–64]. This implies that plants continue to occupy a critical niche in the environment due to their rich possession of secondary metabolites (phytochemicals) that have potential to be used as medicines for several ailments that affect man. Therefore, the use of herbal medicines in the provision of primary health care remains an integral component of all health systems globally [14].

**Table 1:** Medicinal plants used in treatment of symptoms of TB in East Africa

Botanical name	Family	Local Names	Habit	Part used	Country	Author (s)
*Acacia ataxacantha* DC	Fabaceae	Not reported	Tree	Roots	Kenya	[28]
*Acacia hockii* De Wild.	Fabaceae	Kasana (Luganda), Kashiono	Tree	Leaves, Stem bark	Uganda	[7, 10]
*Acacia horrida* (L.)	Fabaceae	Lerai (Samburu)	Tree	Stem bark	Kenya	[29]
*Acacia mearnsii* De Wild.	Fabaceae	Burikoti	Tree	Stem bark	Uganda	[10]
*Acacia nilotica* (L.) Willd. Ex Delile	Fabaceae	Sunut	Tree	Fruit	South Sudan	[30]
*Acacia polyacantha* Willd.	Fabaceae	Egirigirioi	Tree	Stem bark	Uganda	[10]
*Acacia senegal*	Fabaceae	Lderekesi (Samburu)	Tree	Stem bark	Kenya	[29]
*Acacia spectabilis* A. Cunn. Ex Benth.	Fabaceae	Gasiya (Luganda)	Tree	Leaves	Uganda	[7]
*Acanthus pubescens* (Thomson ex Oliv.) Engl.	Acanthaceae	Matovu, Itojo	Herb	Roots	Uganda, Kenya	[10, 12]
*Achyranthes aspera* L.	Amaranthaceae	Muhurura	Herb	Flower	Uganda	[10]
*Achyrospermum carvalhoi* Gürke	Lamiaceae	Kanyamafundo	Shrub	Leaves	Uganda	[10]
*Acokanthera friesiorum*	Apocynaceae	Chipilikwa (Samburu)	Tree	Leaves	Kenya	[29]
*Adenia gummifera*	Passifloraceae	Chepnyalildet (Nandi)	Climber	Roots	Kenya	[31]
*Adhatoda engleriana* Lindau C.B. Clarke	Acanthaceae	Iringoringo (Chagga)	Herb	Roots	Tanzania	[32]
*Ageratum conyzoides* L.	Asteraceae	Namirembe (Luganda)	Herb	Whole plant	Uganda	[7]
*Alangium chinense* (Lour.) Harms	Cornaceae	Omusiisa (Luganda)	Herb	Stem bark	Uganda	[7]
*Albizia anthelmitica*	Fabaceaa	Lamurtana (Samburu)	Tree	Stem bark	Kenya	[29]
*Albizia coriaria* Welw. Ex Oliv	Fabaceae	Mugavu (Luganda), Etek (Lango), Musita (Lusoga), Omusesa (Runyangkore), Omubele (Wanga)	Tree	Stem bark	Uganda, Kenya	[7–10, 12, 33]
*Albizia* species	Fabaceae	Ennongo (Luganda)	Tree	Stem bark	Uganda	[7]
*Albizia versicola*	Fabaceae	Not reported	Tree	Leaves	Tanzania, Kenya	[12]
*Albizia zygia* (DC.) Macbr.	Fabaceae	Ekegonchori (Kuria)	Tree	Roots	Kenya	[12]
*Allium sativum* L.	Alliaceae	Kitungu saumu (Luo), Garlic (Luganda)	Herb	Leaves	Uganda, Kenya	[10, 12]
*Aloe vera* (L.) Burm. f.	Asphodelaceae	Kigaji (Luganda)	Herb	Leaves	Uganda	[7]
*Aloe secundiflora* Engl.	Aloaceae	Sukuroi (Samburu), Osukuroi (Masai), Kiluma (Kamba)	Herb	Leaves	Kenya	[12, 34]
*Amaranthus spinosus*	Amaranthaceae	Kidodo (Luganda)	Herb	Leaves	Uganda	[10]
*Anogeissus leiocarpus* (DC.) Guill. & Perr.	Combretaceae	Sahab	Tree	Stem bark	South Sudan	[30, 35]
*Antiaris toxicaria* Lesch.	Moraceae	Kirundu (Luganda)	Tree	Stem bark	Uganda	[7]
*Asparagus africanus* Lam.	Asparagaceae	Mukira gwango (Luganda)	Climber	Stem bark	Uganda	[10]
*Aspilia africana* (Pers.) C.D. Adams	Asteraceae	Makaayi (Luganda) Emaruoit	Herb	Root bark, Leaves	Uganda	[7, 10]
*Aspilia pluriseta* Schweinf.	Asteraceae	Rirangera	Herb	Roots	Kenya	[28]
*Azadirachta indica* L.	Meliaceae	Muarubaini (Kamba)	Tree	Seeds	Kenya	[12]
*Azadirachta indica* A. Juss.	Meliaceae	Neem tree (Luganda)	Tree	Leaves, stem bark	Uganda	[7, 10]
*Balanites aegyptiaca* (L.) Delile	Zygophyllaceae	Olngosua (Maasai), Ekorete	Shrub	Stem bark	Tanzania, Kenya; Uganda	[10, 12]
*Bersama abyssinica* Fres.	Melianthaceae	Kipsigriet (Sabaot), Kibuimetiet (Nandi)	Tree	Leaves	Kenya	[36]
*Bidens pilosa* L.	Asteraceae	Sere, Labika (Luganda), Kalala (Lusoga), ononot (Lango)	Herb	Flowers, Leaves	Uganda, Rwanda, Burundi	[7, 10, 37, 38]
*Blighia unijugata* Baker	Sapindaceae	Enkuza nyana (Luganda)	Tree	Stem bark	Uganda	[7]
*Boscia senegalensis* (Pers.) Lam.	Capparaceae	Kursan; Mukheit	Shrub	Not reported	South Sudan	[35]
*Bridelia micrantha* (Hochst.) Baill.	Euphorbiaceae	Katazamitti (Luganda), Umugimbu,	Tree	Stem bark, Root	Uganda, Burundi	[7, 38]
*Brillantaisia owariensis* P. Beauv.	Acanthaceae	Icuga	Herb	Leaves	Uganda	[10]
*Cadaba farinosa* Forssk	Capparaceae	Lumuriai (Samburu), Akado marateng (Luo)	Shrub	Not reported	Kenya	[39]
*Callistemon citrinus* (Curtis) Skeels	Myrtaceae	Mwabalabutonya (Luganda)	Shrub	Leaves, Stem bark	Uganda	[7, 9, 10]
*Canarium schweinfurthii* Engl.	Burseraceae	Muwafu (Luganda), Mubafu (Lusoga, Rutoro)	Tree	Stem bark, stem, roots	Uganda, Kenya	[7, 9, 12]
*Canephora pierre* ex A. Froehner	Rubiaceae	Emwanyi (Luganda)	Shrub	Stem bark	Uganda	[7]
*Capparis erythrocarpos* Isert	Capparaceae	Muzingani omwelu, Kitunku ekitono	Shrub	Roots	Uganda	[10]
*Capparis tomentosa* Lam.	Capparaceae	Muzingani omwelu, Kitunku ekitono	Shrub	Roots	Uganda	[10]
*Carica papaya* L.	Caricaceae	Amapapali, Paapali essajja (Luganda), Mupapali omusaiza (Lusoga), Apapalu (Lango)	Shrub	Leaves, Stem	Uganda	[7, 9, 10]
*Carissa edulis* (Forsk.) Vahl	Apocynaceae	Muyonza, Ekamuriei (Ateso)	Shrub	Roots	Uganda	[10]
*Cassine buchananii* Loes.	Celastraceae	Mbaluka (Luganda)	Tree	Stem bark, Leaves	Uganda	[8]
*Catha edulis* Forsk*.*	Celastraceae	Chemgangoi (Sabaot)	Shrub	Stem bark	Kenya	[36]
*Celosia trigyna* L.	Amaranthaceae	Kakubaggiri (Luganda)	Herb	Leaves	Uganda	[7]
*Chaetacme aristata* Planch*.*	Ulmaceae	Embutami (Luganda)	Tree	Leaves	Uganda	[7]
*Cinnamomum zeylanicum* Blume	Lauraceae	Mudalasini (Luganda)	Tree	Stem bark	Uganda	[7]
*Cissampelos pereira* L.	Menispermaceae	Karigi munana	Liana	Roots	Kenya	[28]
*Cissus quinquangularis* L.	Vitaceae	Sukurtuti	Herb	Roots	Kenya	[12, 34]
*Citrus limon* (L.) Osbeck	Rutaceae	Nimawa	Tree	Fruit	Uganda	[9]
*Combretum molle* R.Br. ex. G. Don.	Combretaceae	Ndagi, Loro (Lango)	Tree	Stem bark	Uganda	[7, 8, 10]
*Commiphora* species	Burseraceae	Oltemuai (Sabaot)	Shrub	Not reported	Kenya	[40]
*Commiphora edulis (*Klotzsch*)*	Burseraceae	Not reported	Shrub	Stem bark, Leaves	Kenya	[12, 26]
*Commiphora ellenbeckii* Engl*.*	Burseraceae	Not reported	Shrub	Stem bark, Leaves	Kenya	[26]
*Commiphora mildbraedii* Engl.	Burseraceae	Not reported	Shrub	Stem bark, Root bark	Kenya	[26]
*Cordia africana* Lam	Boraginaceae	Not reported	Tree	Roots	Tanzania, Kenya	[12]
*Crassocephalum vitellinum*	Apiaceae	Akayungubira	Herb	Leaves	Burundi	[38]
*Crossopteryx febrifuga* (Afzel. ex G.Don) Benth.	Rubiaceae	Not reported	Tree	Roots	Tanzania, Kenya	[12]
*Croton dichogamus* Pax.	Euphorbiaceae	Oloiborrbenek (Massai)	Shrub	Roots	Tanzania, Kenya	[12]
*Croton macrostachyus* Hochst. ex Del	Euphorbiaceae	Omutswitswi (Wanga), Mukinduri (Kikuyu)	Tree	Leaves, Roots	Kenya	[33]
*Croton sylvaticus*	Euphorbiaceae	Not reported	Tree	Roots	Tanzania	[41]
*Croton zambesicus*	Euphorbiaceae	Um-Gilagla	Tree	Fruit	South Sudan	[42, 43]
*Cryptolepis sanguinolenta*	Apocynaceae	Kafulu (Luganda)	Shrub	Roots	Kenya, Uganda	[12, 44]
*Cymbopogon citratus* D.C. ex Stapf	Poaceae	Kisubi (Luganda), Akisube (Ateso), Lum cai (Lango)	Herb	Leaves	Uganda	[7]
*Cyperus latifolius* Poir.	Cyperasaceae	Ekekeriaut	Herb	Roots	Uganda	[10]
*Cyperus rotundus* L. Subsp. rotundus	Cyperasaceae	Ekekeriaut	Herb	Roots	Uganda	[10]
*Cyphostemma adenocaule*	Vitaceae	Lordo (Samburu)	Herb	Not reported	Kenya	[34]
*Dalbergia melanoxylon* Guill. & Perr*.*	Fabaceae	Not reported	Tree	Stem bark	Kenya	[28]
*Datura stramonium*	Solanaceae	Not reported	Herb	Leaves	Rwanda	[45]
*Desmodium salicifolium* (Poir.) D.C.	Fabaceae	Enkolimbo (Luganda)	Herb	Leaves	Uganda	[7]
*Desmodium repandum* (Vahl) DC.	Papilionaceae	Ituza	Herb	Leaves	Uganda	[10]
*Dichrostachys cinerea* (L.) Wight and Arn	Fabaceae	Chinjiri (Digo)	Tree	Roots	Kenya	[28]
*Dodonaea angustifolia* L. f.	Sapindaceae	Musambya (Luganda)	Shrub	Leaves	Uganda	[10]
*Dracaena steudneri* Engl.	Asparagaceae	Kajjolyenjovu (Luganda)	Tree	Stem bark	Uganda, Kenya	[7, 9, 10, 12]
*Dychrostachys glomerata* (DG) (Forssk.)	Fabaceae	Not reported	Tree	Leaves, Roots	Uganda, Kenya, Tanzania	[10, 12, 29]
*Embelia schimperi* Vatke	Myrsinaceae	Sachuonet (Ogiek)	Tree	Stem bark	Kenya	[46]
*Entada abbysinica* A. Rich.	Fabaceae	Laginaria (Luo) Mwolola (Luganda)	Shrub	Roots, Stem bark, Leaves	Uganda, Kenya, Tanzania	[7, 10, 12, 29]
*Erythrina abyssinica* Lam. ex DC*.*	Fabaceae	Ejjirikiti (Luganda), Kiko Omoko (Rutoro), Oluo (Lugbara), Owila kot (Lango), Muyirikiti (Lusoga), Omotembe (Kisii)Muhuti (Kikuyu), Umurinzi	Tree	Stem bark, leaves	Uganda, Kenya, Tanzania, Rwanda, Burundi	[7–10, 12, 38, 45, 47]
*Eucalyptus* species	Myrtaceae	Kalintusi (Luganda)	Tree	Leaves, Stem bark	Uganda, Kenya, Tanzania, Rwanda	[7–10, 12, 47, 48]
*Euclea divinorum* Hiern	Ebenaceae	Emus, Kasalagala/Muda (Lusoga)	Shrub	Roots	Uganda	[10]
*Euphorbia ingens* E.Mey. ex Boiss.	Euphorbiaceae	Not reported	Tree	Roots	Kenya	[28]
*Euphorbia schimperiana* Scheele	Euphorbiaceae	Kazagamira (Luganda)	Tree	Leaves	Uganda	[7]
*Faidherbia albida* (Del.) Chevi.	Fabaceae	Haraz	Tree	Leaves	South Sudan	[42]
*Ficus glumosa* Delile	Moraceae	Muwo (Luganda)	Shrub	Stem bark	Uganda	[7]
*Ficus natalensis* Hochst*.*	Moraceae	Omutuba (Luganda), Mugaire (Lusoga)	Tree	Stem bark	Uganda	[7]
*Ficus platyphylla* Delile	Moraceae	Mudodwe	Shrub	Stem bark	Uganda	[10]
*Ficus saussureana*	Moraceae	Omuwo (Luganda)	Shrub	Stem bark	Uganda	[8]
*Fleurya aestuans* (L.) Gaudich. ex Miq.	Urticaceae	Munyango (Luganda)	Herb	Leaves	Uganda	[7]
*Garcinia buchananii* Baker	Clusiaceae	Musaali (Luganda)	Tree	Stem bark, Root bark	Uganda, Kenya, Tanzania	[7, 10, 12]
*Gnaphalium purpureum* L.	Asteraceae	Omuya (Luganda)	Herb	Leaves	Uganda	[7]
*Gnidia buchananii* Gilg	Thymelaeaceae	Not reported	Herb	Roots	Kenya	[49]
*Gomphocarpus physocarpus* E. Mey.	Apocynaceae	Gashaho	Herb	Leaves	Uganda	[10]
*Gutenbergia cordifolia* Benth. ex Oliv*.*	Asteraceae	Ekoutapem	Herb	Roots, Leaves	Uganda	[10]
*Harrisonia abyssinica* Oliv.	Simaroubaceae	Mutagataga (Meru), Osiro (Luo), Orongoriwe (Kuria), Lushaike	Shrub	Stem bark	Uganda, Kenya	[10, 50, 51]
*Harungana madagascariensis* Lam.ex Pior	Hypericaceae	Mukabiiransiko (Luganda)	Tree	Stem bark, Leaves	Uganda	[8]
*Helichrysum odoratissimum* (L.)	Asteraceae	Lweza (luganda)	Herb	Leaves	Uganda	[10]
*Heterotis canescens*	Melastomataceae	Umusomaw’a-bungere,	Herb	Leaves	Burundi	[38]
*Hibiscus fuscus* Garcke	Malvaceae	Lusaala (Luganda)	Herb	Leaves	Uganda	[7]
*Hoslundia opposita* Vahl	Lamiaceae	Cheroronit, Cherungut (Nandi), Nfodo (Lusoga)	Shrub	Leaves	Uganda, Kenya	[10, 31]
*Hypericum revolutum* Vahl	Clusiaceae	Mushungwa	Tree	Leaves	Uganda	[10]
*Hypoestes verticillaris* (L.f.) Sol.	Acanthaceae	Narubat (Ogiek)	Herb	Roots	Kenya	[46]
*Iboza multiflora* (Benth.) E. A. Bruce	Lamiaceae	Iseja	Shrub	Leaves	Uganda	[10]
*Iboza riparia* (Hochst.) N. E. Br.	Lamiaceae	Muravumba	Shrub	Leaves	Uganda	[10]
*Indigofera emarginella* Steud. ex A. Rich.	Fabaceae	Olutunga nsonzi (Luganda)	Shrub	Leaves, Stem bark	Uganda	[7]
*Indigofera lupatana* Baker F	Fabaceae	Not reported	Shrub	Roots	Kenya	[28]
*Kalanchoe glaucescens* Planch. ex Benth	Crassulaceae	Ekiyondo ekyeru (Luganda)	Herb	Leaves	Uganda	[7, 9]
*Kalanchoe integra*	Crassulaceae	Not reported	Shrub	Leaves	Rwanda	[48]
*Khaya senegalensis*	Meliaceae	Not reported	Tree	Leaves, Stem bark	South Sudan	[52]
*Lagenaria sphaerica* (Sond.) Naudin	Cucurbitaceae	Mutanga	Herb	Leaves	Uganda	[10]
*Lantana camara* L.	Verbenaceae	Kayukiyuki (Luganda), Owinybilo (Lango), Kanpanga (Ateso)	Shrub	Leaves	Uganda	[7, 10, 53]
*Lantana trifolia*	Verbenaceae	Not reported	Shrub	Leaves	Rwanda	[48]
*Leonotis nepetifolia* (L.) R. Br.	Lamiaceae	Susuni	Shrub	Leaves	Uganda	[10]
*Leucas calostachys* Oliv.	Lamiaceae	Kakuba musulo (Luganda)	Shrub	Leaves, Whole plant	Uganda	[8]
*Lippia grandifolia* Hochst. ex A. Rich	Verbenaceae	Olugumaguma (Luganda)	Herb	Leaves	Uganda	[7]
*Lonchocarpus eriocalyx* Harms	Fabaceae	Not reported	Tree	Stem bark	Kenya	[11, 28]
*Maesa lanceolata* Forssk.	Myrsinaceae	Muhanga	Tree	Roots	Uganda	[10]
*Mangifera indica* L.	Anacardiaceae	Muyembe (Luganda), Aeme (Lango)	Tree	Stem bark	Uganda, Kenya	[7, 9, 10, 12, 47]
*Maytenus senegalensis* (Lam.)	Celastraceae	Naligwalimu (Luganda), Muwaiswa, Eterka, Itereka (Lango)	Shrub	Root bark, Leaves	Uganda	[7, 10]
*Microglossa pyrifolia* (Lam.)	Asteraceae	Kabilili akatono (Luganda)	Shrub	Roots	Uganda	[10]
*Microgramma lycopodiodes* (L.) Copel	Polypodiaceae	Kukumba (Luganda)	Herb	Roots, Leaves	Uganda	[8]
*Milicia excelsa* (Welw.) C.C. Berg	Moraceae	Muvule (Luganda)	Tree	Leaves	Uganda	[7]
*Momordica foetida* Schumach.	Cucurbitaceae	Bombo (Luganda), Luiwula/Mwishwa	Herb	Leaves	Uganda, Rwanda	[7, 10, 45]
*Momordica rostrata* A. Zimm.	Cucurbitaceae	Chepkologolio (Ogiek)	Herb	Roots	Kenya	[46]
*Morella kandtiana* (Engl.) Verdc. & Polhill	Myricaceae	Mukikimbo (Luganda)	Herb	Roots, Leaves, Whole plant	Uganda	[8]
*Morinda lucida* Benth.	Rubiaceae	Kabaja nsayi (Luganda)	Tree	Stem bark	Uganda	[7]
*Moringa oleifera* Lam.	Moringaceae	Moringa (Luganda)	Tree	Fruit, Stem	Uganda	[7, 10]
*Mucuna pruriens* (L.) DC.	Papilionaceae	Lugenyu (Luganda)	Vine	Leaves	Uganda	[10]
*Myrica kandtiana* Engl*.*	Myricaceae	Enkikimbo(Luganda)	Tree	Fruit, Leaves, Stem bark, Root bark	Uganda	[7]
*Myrsine africana* L.	Myrsinaceae	Seketeti (Samburu)	Shrub	Not reported	Kenya	[34]
*Nauclea latifolia Sm*	Rubiaceae	Karmadoda	Tree	Fruit	South Sudan	[54]
*Ocimum basilicum*	Lamiaceae	Umusurasura	Herb	Leaves	Burundi	[38]
*Ocimum suave* Willd*.*	Lamiaceae	Muhumuzanganda (Luganda)	Herb	Leaves	Uganda	[10]
*Olea capensis* L.	Oleaceae	Pekeriondet (Sabaot)	Tree	Stem bark	Kenya	[36]
*Olinia rochetiana*	Penaeaceae	Kaptolongit (Sabaot)	Tree	Roots	Kenya	[36]
*Ormocarpum trichocarpum* (Taub.) Harms	Papilionaceae	Eseperuae	Tree	Roots	Uganda	[10]
*Pappea capensis* (Spreng) Eckl. & Zeyh.	Sapindaceae	Muba (Kikuyu), Enkorrirri, Oltimigomi (Maasai)	Shrub	Stem bark, Root bark	Kenya	[55, 56]
*Parinari curatellifolia* Planch. ex Benth.	Chrysobalanaceae	Umunazi	Tree	Stem bark, roots	Burundi	[38]
*Pavetta crassipes* K. Schum.	Rubiaceae	Not reported	Shrub	Roots	Tanzania, Kenya	[12]
*Pentas longiflora* Oliv.	Rubiaceae	lsagara	Herb	Roots	Rwanda	[37]
*Persea americana* Mill.	Lauraceae	Ovacado (Luganda)	Tree	Stem bark	Uganda	[7, 9]
*Phaseolus lunatus* L.	Fabaceae	Kayindiyindi (Luganda)	Herb	Leaves	Uganda	[7]
*Phaseolus vulgaris* L.	Fabaceae	Bijanjaro (Luganda)	Herb	Husks	Uganda	[7]
*Phyllanthus reticulatus* Poir*.*	Phyllanthaceae	Mutulika (Luganda)	Shrub	Leaves	Uganda	[7]
*Piliostigma thonningii*	Fabaceae	Chebutiandet (Sabaot)	Tree	Leaves	Kenya	[36]
*Piptadenistrum africana*	Fabaceae	Mpewere (Luganda)	Tree	Stem bark	Uganda	[7, 9, 10]
*Plectranthus barbatus* Andrews	Lamiaceae	Ekibankulata (Luganda), Ebiriri omutano (Ateso)	Shrub	Leaves	Uganda	[7, 10]
*Plectranthus hadiensis*	Lamiaceae	Kibwankulanta (Luganda)	Shrub	Whole plant, Leaves	Uganda	[8]
*Plumbago dawei*	Plumbaginaceae	Lkiarianthus (Samburu)	Herb	Stem bark	Kenya	[29]
*Plumbago zeylanica* L.	Plumbaginaceae	Musajjabanda (Luganda), Mukya (Kamba)	Herb	Leaves	Uganda, Kenya	[7, 34, 57]
*Podocarpus usambarensis* Pilg.	Podocarpaceae	Kamusenene (Luganda)	Tree	Leaves	Uganda	[7]
*Prunus africana* (Hook.f.) Kalkman	Rosaceae	Ntaseesa, Ngwabuzito (Luganda, Rutoro),Sirumandu (Lugisu)	Tree	Stem bark	Uganda	[7]
*Pseudospondia microcarpa* (A. Rich.) Engl.	Anacardiaceae	Muziru (Luganda)	Tree	Stem bark	Uganda	[7]
*Psidium guajava* L.	Myrtaceae	Mpera (Chagga)	Tree	Fruit, Leaves, Stem bark, Root bark	Uganda, Kenya, Tanzania	[7, 12]
*Pycnostachys ericirosenii* R.E.Fr.	Lamiaceae	Musindikwa (Luganda)	Shrub	Leaves	Uganda	[10]
*Rhamnus prinoides* L’Herit.	Rhamnaceae	Munanira (Luganda)	Shrub	Leaves	Uganda	[10]
*Rhoicissus tridentata* (L.f.) Wild. & R.B.D. Drumm.	Vitaceae	Mumara (Luganda)	Shrub	Leaves	Uganda	[10]
*Rhus natalensis* Bernh. ex Krauss	Anacardiaceae	Lmisigiyoi, Muthigiu (Kikuyu)	Tree	Roots, Leaves	Kenya	[51]
*Rhus vulgaris* Meikle	Anacardiaceae	Kakwansokwanso (Luganda)	Herb	Stem bark, Leaves	Uganda	[7]
*Ribes uva-crispa* L.	Grossulariaceae	Entuntunu (Luganda)	Shrub	Leaves	Uganda	[7]
*Rosmarinus officinalis* L.	Lamiaceae	Not reported	Herb	Leaves	South Sudan	[52]
*Rubia cordifolia* L.	Rubiaceae	Kasalabakesi (Luganda) Urumurwa (Kuria)	Herb	Leaves, Whole plant	Uganda, Kenya, Tanzania	[7, 9, 10, 12, 16]
*Rumex abyssinicus* Jacq.	Polygonaceae	Not reported	Herb	Leaves	Rwanda	[48]
*Sapium ellipticum* (Hochst.) Pax	Euphorbiaceae	Omusasa (Luganda)	Shrub	Stem bark	Uganda	[7]
*Securidaca longipedunculata* Fresen.	Polygalaceae	Mukondwa, Awee ilila (Lango), Mukondwa (Lusoga), Eliloi (Ateso)	Tree	Roots	Uganda	[8, 10]
*Senna siamea* (Lam.) Irwin & Barneby	Fabaceae	Gasiya seed	Tree	Stem bark	Uganda	[10]
*Sesamum calycinum*	Pedaliaceae	Lutungotungo (Luganda)	Herb	Leaves, Whole plant	Uganda	[8]
*Solanum aculeastrum* Dunal	Solanaceae	Mutura (Kikuyu), Ekitengo (Luganda)	Shrub	Fruit, Roots, Leaves	Uganda, Kenya	[7, 8, 12]
*Solanum incanum* L.	Solanaceae	Entengotengo Ennene (Luganda), Ocokocok (Lango), Ntonka (Lusoga), Mutongu (Kamba),Entulelei (Maasai)	Shrub	Fruit	Uganda, Kenya	[7, 12]
*Solanum mauense* Bitter	Solanaceae	Ng’onyoyiek (Ogiek)	Shrub	Seeds	Kenya	[46]
*Spathodea campanulata* P. Beauv.	Bignoniaceae	Kifabakazi (Luganda)	Tree	Stem bark	Uganda	[7]
*Syzygium cumini* (L.) Skeels	Myrtaceae	Jambula (Luganda)	Tree	Stem bark	Uganda	[7, 9]
*Tamarindus indica* L.	Fabaceae	Mukoge (Luganda), Cwao (Lango)	Tree	Leaves	Uganda	[10]
*Teclea nobilis* Del.	Rutaceae	Luzo	Shrub	Leaves	Uganda	[10]
*Tetradenia riparia* (Hochst.) Codd	Lamiaceae	Ekyewamala (Luganda)	Herb	Leaves	Uganda, Rwanda	[7, 37]
*Terminalia laxiflora* Engl. & Diels	Combretaceae	Darout	Tree	Stem bark	South Sudan	[30]
*Tithonia diversifolia* (Hemsl.) A. Gray	Asteraceae	Ekimyula, Okelokelo (Lango)	Shrub	Stem bark	Uganda	[7]
*Toddalia asiatica* (L.) Lam	Rutaceae	Simborichet (Sabaot), Mururue (Kikuyu), Oleparmunyo (Maasai), Kawule (Luganda)	Shrub	Roots, Leaves	Uganda, Kenya	[7, 8, 10, 36]
*Tragia brevipes* Pax	Euphorbiaceae	Nakepian	Climber	Roots	Uganda	[10]
*Tragia subsessilis* Pax	Euphorbiaceae	Totoananyia	Herb	Roots	Uganda	[10]
*Trichilia dregeana* Sond.	Meliaceae	Sekoba (Luganda)	Tree	Stem bark,	Uganda	[7]
*Triumfetta flavescens* Hochst. ex A. Rich.	Malvaceae	Luwugula (Luganda)	Shrub	Stem	Uganda	[7]
*Vachellia drepanolobium* (Harms ex Sjostedt) P.J.H. Huter	Fabaceae	Oluai (Maasai)	Tree	Stem bark, Root bark	Kenya	[55]
*Vernonia cinerea* (L.) Less.	Asteraceae	Kayayana, Lukohe (Luganda), Yat Kwong (Lango)	Herb	Leaves	Uganda	[7]
*Vernonia amygdalina* Del.	Asteraceae	Mululuza (Luganda) Lubilili	Shrub	Leaves	Uganda	[7, 10]
*Warburgia ugandensis* Sprague	Canellaceae	Abaki, Sokoni (Samburu), Muthiga (Kikuyu)	Tree	Stem bark	Uganda, Kenya, Tanzania	[7–10, 12, 16, 57–59]
*Zanthoxylum chalybeum* Engl.	Rutaceae	Ntale ya ddungu (Luganda), Eusuk (Ateso), Agodaman (Lango), Oloisuki (Maasai), Rukuts (Karimojong), Outiku (Lugbara)	Tree	Stem bark	Uganda, Kenya, Tanzania	[5, 8–10, 12]
*Zanthoxylum gillettii* (De Wild.) P.G. Waterman	Rutaceae	Sagawatiet, Shihumba/Shikuma	Tree	Stem bark	Kenya	[31]
*Zanthoxylum leprieurii*	Rutaceae	Not reported	Tree	Stem bark	Uganda	[5]
*Zehneria scabra*	Cucurbitaceae	Umushishiro,	Herb	Leaves	Burundi	[38]
*Zingiber officinale*	Zingiberaceae	Tangawizi (Luo), Ntangawuzi (Luganda)	Herb	Stem	Uganda, Kenya	[7, 9, 10, 12]

Languages: Ateso, Lango, Luganda, Lugbara, Lugisu, Lusoga, Karimojong, and Rutoro (Uganda); Digo, Kamba, Kikuyu, Kisii, Kuria, Luo, Maasai, Meru, Nandi, Ogiek ,Sabaot, Samburu, and Wanga (Kenya); and Chagga (Tanzania). Local names with language(s) not indicated were not specified by the authors

**Fig. 1:**
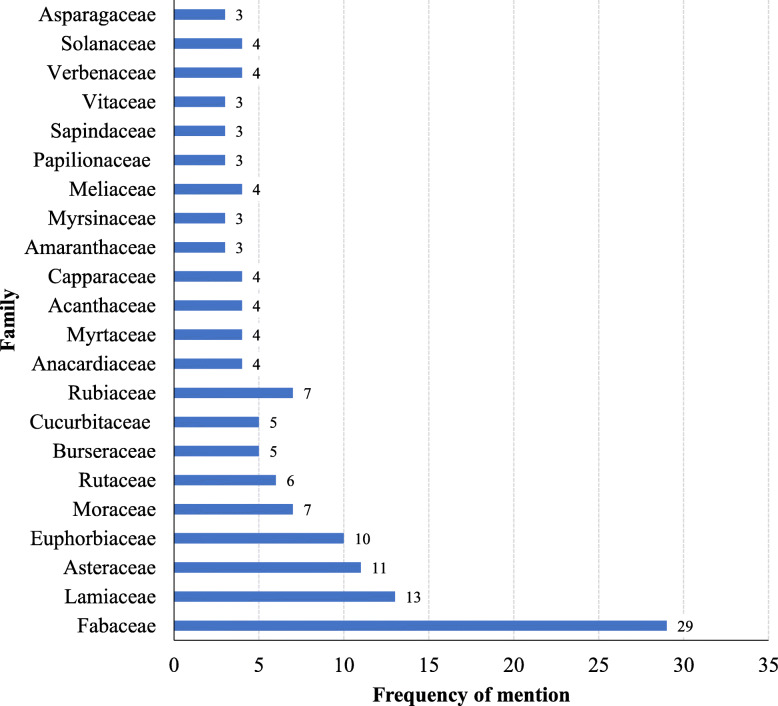
Major botanical families from which TB remedies are obtained in East Africa

**Fig. 2:**
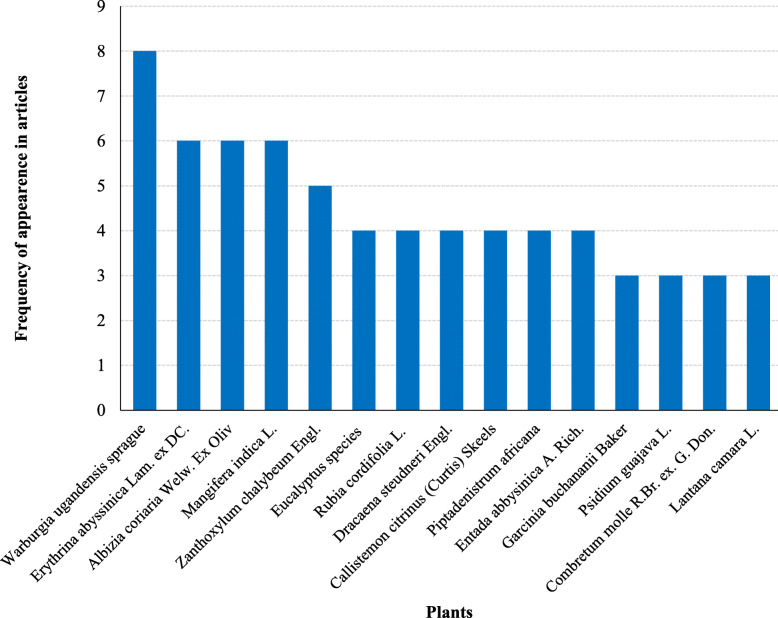
The most cited plant species used for treatment of TB and its symptoms in East Africa

Most encountered species were from the family Fabaceae (42.6%), Lamiaceae (19.1%), Asteraceae (16.2%), Euphorbiaceae (14.7%), Moraceae (10.3%), Rubiaceae (10.3%), Rutaceae (8.8%), Burseraceae (7.4%), and Cucurbitaceae (7.4%) (Fig. 1). Fabaceae, Asteraceae, and Lamiaceae were also reported to provide the largest number of plants species used for TB management in South Africa, Ghana, Nigeria, Ethiopia, and India [64–72]. From these families, 15 species were the most cited in East Africa (Fig. 2). These families were reported from at least four countries of East Africa. This could probably be attributed to the abundant distribution of the analogue active substances among species from these families [23, 24]. The family Fabaceae has biosynthetic pathways that produce majorly flavonoids, terpenoids, and alkaloids as secondary metabolites [73–75]. It is these phytochemicals that are responsible for the antimycobacterial activity against different mycobacterial strains [67, 70, 76, 77]. Other families reported in East Africa to house medicinal plants for management of TB and have also been reported in other countries include Acanthaceae, Apocynaceae, Cariaceae, Combretaceae, Malvaceae, Moraceae, Myrtaceae, Rhamnaceae, Rubiaceae, Solanaceae, and Zingiberaceae [64, 72, 78–81].

**Fig. 3:**
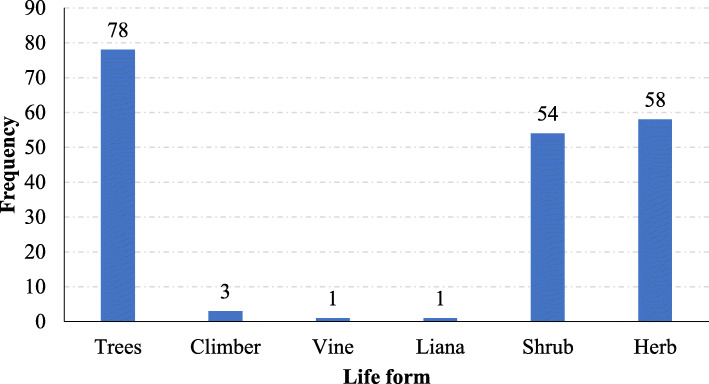
Growth habit of the plants used for preparation of antitubercular remedies in East Africa

Geographically, none of the documented plant species was reported to be used in the management of TB across all the East African countries. However, two plant species (*Erythrina abyssinica* and *Eucalyptus* species) are used by at least 4 countries. A total of 30 plant species were reported to be used by at least two countries. Uganda had the highest number of species mentioned followed by Kenya and then Tanzania (Table 1). The differences in species utilization could be attributed to the differences in soil chemistry, rainfall, topography, and climate that results into differences in phytochemical composition of the same species growing in different geographical areas [82]. Additionally, it could also be due to differences in knowledge and experiences as result of different social and cultural backgrounds that exists across the countries. Uganda had many ethnobotanical surveys conducted to document medicinal plants used in the management of tuberculosis as compared to other countries. Most of these medicinal plants were growing as trees (40.0%), herbs (29.7%), shrubs (27.7%), and rarely as climbers, vines, or lianas (Fig. 3).

**Fig. 4:**
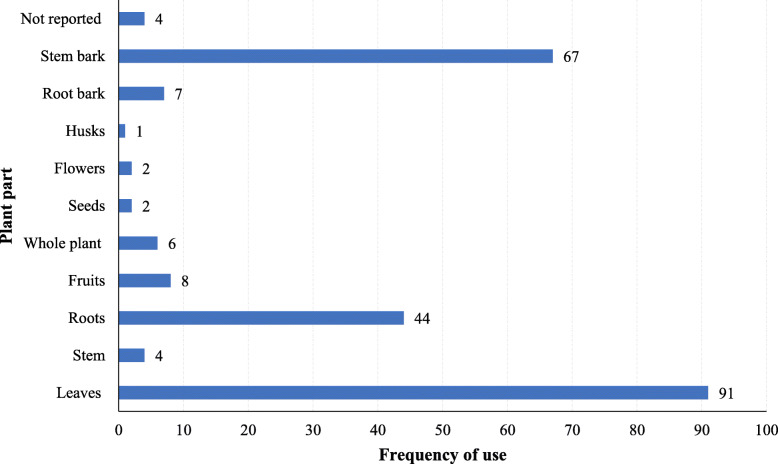
Frequency of plant parts used for preparation of antitubercular remedies in East Africa

Analysis of ethnomedicinal recipes revealed that mainly leaves (38.6%), stem bark (28.4%), and roots (18.6%) were used for preparing herbal remedies. Root bark, whole plants, fruits, flowers, seeds, and husks were rarely used (Fig. 4). Harvesting of leaves and stem bark allows sustainable utilization of the plants hence promoting their conservation as opposed to use of roots and whole plants. Additionally, leaves are the primary sites for secondary metabolic pathways in plants while stem barks act as major concentration areas (deposition sites) for the synthesized metabolites [9, 57].

Most articles reviewed reported that traditional herbal medicine practitioners usually combined different plant species while preparing herbal medicines. However, they did not report how the herbal medicine from individual plant species can be prepared. Decoction was by far the commonest method of herbal medicine preparation cited. Others included cold infusions, drying and pounding into a powder, burning into ash, chewing, and steaming. Use of more than one plant in combination is more effective than single plant perhaps due to the synergistic interactions that occur among the different phytochemicals that result into increased bioactivity (efficacy). But also, the benefit of phytochemicals from one species counteracting the toxicity of another species could be another explanation.

The major route of administration was oral (via the mouth) although sometimes inhalation and topical application were also reported depending on the preparation method used and the toxicity of the plant(s). Cups, bottles, and tablespoons were the most commonly used for determining the posology of herbal remedies [7, 10, 12].

### Efficacy and safety studies

Some ethnobotanical studies reported that herbal medicine preparations were effective in the treatment of TB, while some were used in the management of multidrug-resistant tuberculosis [7, 12, 47]. This could be due to the synergistic interaction between the various phytochemicals present in the herbal preparations [27, 83]. However, as much as these herbal medicines might have genuine bioactivity, sometimes they are used concurrently with conventional therapies as supplements and at times adulterated. Therefore, it is important to scientifically validate the claimed efficacy and safety of both the herbal preparations and the individual medicinal plants. Out of the 195 species documented, only 36 plant species (18.5%) have been studied for their antimycobacterial activity. A WHO report [14] indicated that only approximately 10% of the world’s flora have been studied as regards their medicinal potential. This has greatly hindered the discovery of potential lead compounds that could be developed into new antitubercular drugs.

Out of the 36 screened medicinal plants, 31 species (86.1%) were reported to be bioactive with some species exhibiting quite considerable antimycobacterial activity although the current standard drugs had superior bioactivity (Table 2). This is comparable to India where 70% of 365 plants which were studied showed antimycobacterial activity [87]. Among the promising plant species (with minimum inhibitory concentration less than 0.5 mg/ml) were *Erythrina abyssinica*, *Entada abyssinica*, *Bidens pilosa*, *Callistemon citrinus*, *Khaya senegalensis*, *Lantana camara*, *Piptadenistrum africana*, *Rosmarinus officinalis*, *Tetradenia riparia*, and *Zanthoxylum leprieurii*. Isolated pure compounds from three of the promising plant species had much higher activity against Mtb than the crude extracts and fractions. Indeed, some of the compounds from *Zanthoxylum leprieurii* had minimum inhibitory concentrations lower than those of standard antitubercular drugs (Table 3). Crude extracts and fractions usually have less pharmacological activity than standard drugs because of the interference from other inactive substances in the matrix that reduce the overall concentration of the active molecules in the tested dose. This explains why isolation of pure compounds is a critical step in natural product drug discovery process. The five documented medicinal plants that were found to be inactive are *Acacia ataxacantha*, *Dalbergia melanoxylon*, *Indigofera lupatana*, *Lonchocarpus eriocalyx*, and *Solanum incanum*. This could probably be attributed to the absence of inherent bioactive phytochemicals against Mtb in the plant species. This could be brought about by absence or impaired biosynthetic metabolic pathways due to unfavorable growth conditions in the habitat from where the plants grow. This implies that herbal remedies for TB containing each of these plants singly may not be effective. Therefore, other benefits provided by these species in the concoctions of TB such as detoxification of other toxic phytochemicals, preservation of the herbal medicine, or potentiation of the pharmacological activity of other phytochemicals could be investigated.

**Table 2:** Efficacy, toxicity, and phytochemical studies on medicinal plants used for treatment of TB in East Africa

Plant	Extraction method (solvent)	MIC (μg/ml) on H37Rv strain	MIC (μg/ml) on TMC-331 strain	Toxicity of crude extracts (μg/ml)	Class of compounds	Author(s)
*Acacia ataxacantha*	Maceration (methanol)	Not active	Not tested	IC_50_ = 90.39	Phenols, terpenoids	[28]
*Acacia horrida*	Soxhlet (methanol)	< 1000 (Iso < 500)	Not tested	Not tested	Alkaloids, cardiac glycosides, tannins, saponins, terpenoids	[29]
*Acacia senegal*	Soxhlet (methanol)	< 1000 (Iso < 500)	Not tested	Not tested	Cardiac glycosides, tannins, saponins, terpenoids, flavonoids	[29]
*Acokanthera friesiorum*	Soxhlet (methanol)	< 1000 (Iso < 500)	Not tested	Not tested	Cardiac glycosides, Tannins, flavonoids	[29]
*Albizia anthelmitica*	Soxhlet (methanol)	< 1000 (Iso < 500)	Not tested	Not tested	Alkaloids, saponins, tannins, flavonoids	[29]
*Aspilia pluriseta*	Maceration (methanol)	Active at 1 g/ml (MIC not determined)	Not tested	IC_50_ = 24.51	Phenol, terpenoids, flavonoids	[28]
*Bidens pilosa*	Maceration (ethanol)	100	Not tested	Not tested	Not tested	[37]
*Callistemon citrinus*	Maceration (methanol, chloroform)	325 (methanol), 48 (chloroform) (Iso = 4.0; R = 2.0)	78 (methanol), 158 (chloroform), Iso = 4.0	Not tested	Flavonoids, alkaloids, triterpenoids, saponins	[15]
*Cissampelos pareira*	Maceration (methanol)	Active at 1 g/ml (MIC not determined)	Not tested	IC_50_ = 179	Anthraquinones, phenols, terpenoids, flavonoids	[28]
*Commiphora edulis*	Maceration (ethyl acetate, DCM, water)	6250 (Ethyl acetate), 780 (methanol), Not active (water)	Not tested	IC_50_ = 393 (DCM), 1734 (ethyl acetate)	Flavonoids, terpenoids	[26]
*Commiphora ellenbeckii*	Maceration (ethyl acetate, methanol, water)	12500 (Ethyl acetate), 3125 (methanol), 780 (water), 15 (rif)	Not tested	IC_50_ = 608 (methanol), 1509 (water)	Alkaloids, saponins, tannins, phenols, flavonoids, terpenoids	[26]
*Commiphora mildbraedii*	Maceration (ethyl acetate, methanol, water)	6250–9250 (Ethyl acetate), 390–780 (methanol), not active (water), 15 (Rif)	Not tested	IC_50_ = 339 (ethyl acetate), 452 (methanol)	Alkaloids, saponins, tannins, phenols, flavonoids, terpenoids	[26]
*Cordia sinensis*	Soxhlet (methanol)	< 500 (Iso < 500)	Not tested	Not tested	Saponins, terpenoids, flavonoids, tannins	[29]
*Cryptolepsis sanguinolenta*	Methanol chloroform	1170 (methanol) (Iso = 0.25; R = 0.25)	1580 (methanol) (Iso = 0.25)	LD_50_ = 758 mg/kg	Alkaloids, tannins, flavonoids	[84]
*Dalbergia melanoxylon*	Maceration (methanol)	Not active	Not tested	IC_50_ = 120.04	Phenols, terpenoids	[28]
*Dichrostachys cinerea*	Maceration (methanol)	Active at 1 g/ml, (MIC not determined)	Not tested	IC_50_ = 201.22	Phenols, terpenoids	[28]
*Entadda abyssinica*	Maceration (methanol)	500 (Iso = 0.25)	Not tested	Not tested	Flavonoid, alkaloids, saponins, tannins	[12, 29]
*Erythrina abyssinica*	Maceration (methanol)	390 (Rif = 0.25; Iso = 0.25)	2350 (Iso = 9.38)	LD_50_ = 776.2 mg/kg	Flavonoids, alkaloids, tannins	[44]
*Euphorbia ingens*	Maceration (methanol)	Active at 1 g/ml (MIC not determined)	Not tested	IC_50_ = 105.55	Phenols, terpenoids	[28]
*Euphorbia scarlatica*	Soxhlet (methanol)	< 500 (Iso < 500)	Not tested	Not tested	Alkaloids, cardiac glycosides, terpenoids, flavonoids	[29]
*Gnidia buchananii*	Maceration (methanol)	Active at 1 g/ml (MIC not determined)	Not tested	IC_50_ = 76.24	Phenols, terpenoids,	[28]
*Indigofera lupatana*	Maceration (methanol)	Not active	Not tested	IC_50_ = 60.37	Phenols, terpenoids	[28]
*Khaya senegalensis*	Maceration (ethyl acetate, chloroform)	6.25	Not tested	IC_50_ = 1000	Not tested	[52]
*Lantana camara*	Maceration (methanol, chloroform)	20 (Rif = 1)	15 (Iso = 0.25)	LD_50_ > 500 mg/kg	Not reported	[53]
*Lonchocarpus eriocalyx*	Maceration (methanol)	Not active	Not tested	IC_50_ = 201.87	Terpenoids, phenols, flavonoids	[28]
*Loranthus acaciae*	Soxhlet (methanol)	< 1000 (Iso < 500)	Not tested	Not tested	Alkaloids, cardiac glycosides, saponins, flavonoids	[29]
*Mangifera indica*	Methanol	3130 (methanol) (Iso = 0.25; R = 0.25)	590 (methanol) (Iso = 0.25)	Not tested	Phenols, terpenoids	[16]
*Pentos longiflora*	Maceration (ethanol)	1000	Not tested	Not tested	Not tested	[37]
*Piptadenistrum africana*	Maceration (chloroform)	395 (chloroform)	395 (chloroform)	Not tested	Flavonoids, tannins	[15]
*Plumbago dawei*	Soxhlet (methanol)	< 1000 (Iso < 500)	Not tested	Not tested	Cardiac glycosides, tannins, terpenoids, flavonoids	[29]
*Rosmarinus officinalis* L.	Maceration (chloroform)	6.25	Not tested	IC_50_ = 100	Not tested	[52]
*Salvadora persica*	Soxhlet (methanol)	< 500 (Iso < 500)	Not tested	Not tested	Alkaloids, cardiac glycosides, terpenoids, flavonoids	[29]
*Solanum incanum*	Methanol chloroform	Not active	Not active	Not tested	Not reported	[16]
*Tetradenia riparia*	Maceration (ethanol)	500	Not tested	Not tested	Not tested	[37]
*Warburgia ugandensis*	Methanol chloroform	4690 (methanol), 2350 (chloroform) (Iso = 0.25; R = 0.25)	2350 (methanol), 590 (chloroform) (Iso = 0.25)	Not tested	Flavonoids, tannins, terpenoids	[85, 86]
*Zanthoxylum leprieurii*	Methanol	47.5 (Iso = 4.0; R = 2.0)	75.3 (Iso = 4.0)	Not tested	Alkaloids	[5]

*IC*_*50*_ median cytotoxic concentration, *LD*_*50*_ median lethal dose, *Iso* isoniazid, *Rif* rifampicin, *H37Rv* pan sensitive Mtb strain, *TMC331* rifampicin-resistant Mtb strain, *MIC* minimum inhibitory concentration. Extracts in [26] were tested against *Mycobacteria smegmatis*

**Table 3:** Isolation and characterization studies on medicinal plants used for management of TB in East Africa

Plant	Pure compounds with antitubercular activity	Chemical class	MIC of pure compounds (μg/ml)	Author(s)
*Zanthoxylum leprieurii*	2-hydroxy-1, 3-dimethoxy-10-methyl-9-acridone (**1**), 1-hydroxy-3-methoxy-10-methyl-9-acridone (**2**), 3-hydroxy-1, 5, 6-trimethoxy-9-acridone (**3**)	Acridone alkaloids	1.5 (**1**), 0.2 (**2**), 0.4 (**3**); tested against H37Rv	[5]
*Warburgia ugandensis* Sprague	Muzigadial (**4**), muzigadiolide (**5**), linoleic acid (**6**)	Sesquiterpenes	64 (**4**), 128 (**5**), 16 (**6**); tested against *M*. *smegmatis*	[58, 85]
*Tetradenia riparia*	15- sandaracopimaradiene-7α, 18-dio1 (**7**)	Diterpenediol	25–100	[37]

*MIC* minimum inhibitory concentration. No toxicity studies of the pure compounds were conducted.

All toxicity studies reviewed evaluated only the acute toxicity profiles of the medicinal plants either in vitro or in vivo but not both. Of the bioactive extracts screened, less than half of them were tested for their acute toxicity. Selectivity index (SI) is used as the best estimate of the relative toxicity of a compound to normal mammalian cells as compared to the pathogen and hence its suitability for being a drug candidate. According to the SI criterion, compounds with higher SI are regarded to have better toxicity profiles than those with lower SI [88]. From the retrieved data, only two plant species (*Khaya senegalensis* and *Rosmarinus officinalis*) had acceptable selectivity indices to warrant drug discovery from them. In this study, the SI of only five plant species could be calculated (Table 4) because they were the only plant species with both the inhibitory concentration on Mtb and cytotoxic concentration on normal mammalian cell lines (IC_50_) reported. Hence, there is need to emphasize dual testing of both toxicity and efficacy of natural products for drug development purposes.

**Table 4:** Selectivity indices of some antitubercular plant species reported in East Africa

Plant	Solvent	MIC on Mtb strain (μg/ml)	IC_50_ (μg/ml)	SI	Comment
*Commiphora edulis*	Dichloromethane	1560	393	0.25	More toxic to human cells than the Mtb; not useful
	Ethyl acetate	3125	1734	0.55	More toxic to human cells than the Mtb; not useful
*Commiphora ellenbeckii*	Water	780	1509	1.93	More toxic to Mtb than human cells but the SI is low. May be optimized for lead candidate identification
	Methanol	3125	608	0.19	More toxic to human cells than the Mtb; not useful
*Commiphora mildbraedii*	Methanol	390	452	1.16	More toxic to Mtb than human cells but the SI is close to 1. No practical application
	Ethyl acetate	6250	339	0.054	More toxic to human cells than the Mtb; not very useful
*Khaya senegalensis*	Chloroform	6.25	1000	160	More toxic to Mtb than human cells with high SI. Promising for development of lead candidate
*Rosmarinus officinalis* L.	Chloroform	6.25	100	16	More toxic to Mtb than human cells with high SI. Promising for development of lead candidate

*IC*_*50*_ cytotoxic concentration normal cells, *SI* selectivity index

Two other systems of acute toxicity classification: The National Cancer Institute (NCI) and Organization for Economic cooperation and development (OECD) guidelines 423 were used to assess the toxicity profiles of the different extracts [89, 90]. There was no single plant species among those tested for acute toxicity that was reported to be highly toxic (with IC_50_ less than 20 μg/ml). All the plant species with promising bioactivity that were tested for toxicity had acceptable acute toxicity profiles. These included *Rosmarinus officinalis*, *Lantana camara*, *Khaya senegalensis*, and *Erythrina abyssinica* (Table 2). *Aspilia pluriseta*, *Cissampelos pareira*, *Euphorbia ingens*, and *Gnidia buchananii* had moderate toxicity with IC_50_ between 20 and 200 μg/ml. According to OECD 2001 guidelines, *Lantana camara*, *Erythrina abyssinica*, and *Cryptolepis sanguinolenta* had slight toxicity as their median lethal doses (LD_50_) were above 500 mg/kg. These results justify the general public belief that traditional medicines are relatively safer as compared to the current conventional therapies. However, toxicity testing should be done on all potential medicinal plants and their phytochemicals before concluding that they are safe for human treatment [91–94]. This is because toxicity of herbal medicines may be due to presence of inherent poisonous chemicals in the plant species, misidentification of the plant species, adulteration or contamination during harvesting, preparation, and storage [95, 96]. Acute toxicity tests determine a single high dose that kills 50% of the cells or animals in a population. They may not be evident enough to depict the real toxicity situation for herbal remedies taken for a longer time in chronic conditions like TB [18, 97]. Therefore, this may necessitate sub-chronic and chronic toxicity tests to be carried out on a medicinal plant species with a potential lead compound [95].

### Phytochemistry of the reported plants

Phytochemical investigation reveals the chemical nature of the pure compounds that are responsible for the pharmacological activity as well as the toxicity of medicinal plants [19, 64, 98–101]. Chromatographic and spectroscopic techniques are used to identify and elucidate the chemical structures of compounds [102–107]. In this study, maceration was the commonly used method of extraction as compared to Soxhlet. Majority of the hexane extracts were reported to be inactive against mycobacterial strains while almost all methanolic extracts were active. Methanol being a polar solvent extracts polar phytochemical while hexane (a non-polar solvent) extracts non-polar compounds. It is reasonable to assert that the antimycobacterial activity of the extracts is largely due to polar phytochemicals. There were variations in bioactivity of different parts of the same plant with no specific patterns. This could be due to differences in their rate of accumulating the bioactive substances.

The phytochemicals that were frequently screened for have been alkaloids, saponins, cardiac glycosides, flavonoids, terpenoids, and phenols. All these secondary metabolites were reported to be present in different bioactive extracts. The most commonly reported phytochemicals were flavonoids, terpenoids, and alkaloids [15, 17, 26, 29, 70, 106, 108]. Flavonoids and alkaloids were reported to be absent in three out of the five inactive plants (Table 2). Out of the 31 bioactive plant species, only three (*Tetradenia riparia*, *Warburgia ugandensis*, and *Zanthoxylum leprieurii*) have been further characterized to identify the pure compounds responsible for their antimycobacterial activity [5, 37, 58, 85] (Table 3). This is attributed to the complexity and the rigorous nature of the process that require extraction, screening, isolation, and characterization [100, 109, 110]. Low extraction yield, compound instability, high costs, low technology especially in developing countries, limited access to advanced chromatographic, and spectroscopic equipment and inadequate funding have made it difficult to undertake herbal medicine research [21, 111, 112]. This is further complicated by the microbiological nature of the Mtb that require bioassays to be conducted in biosafety level 3 laboratories that are not readily available in East Africa [60, 113]. More robust and effective techniques are required to fasten the drug discovery process against TB [3, 77, 92, 114].

**Fig. 5:**
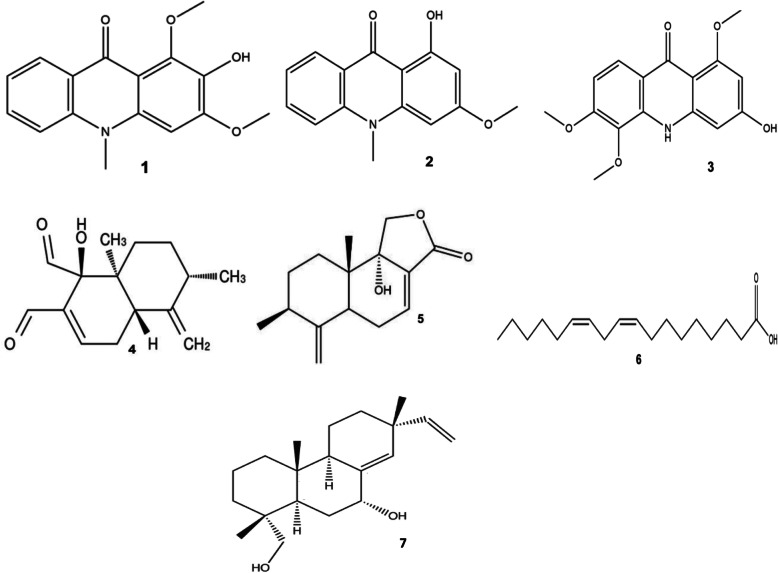
Structure of antitubercular molecules isolated in claimed medicinal plants in East Africa. The numbers **1**–**7** correspond to the molecules mentioned in Table 3

A total of seven pure compounds have been isolated and characterized with bioactivity against Mtb (Fig. 5). These are 2-hydroxy-1,3-dimethoxy-10-methyl-9-acridone (1), 1-hydroxy-3- methoxy-10-methyl-9-acridone (2), 3-hydroxy-1, 5, 6-trimethoxy-9-acridone (3), muzigadial (4), muzigadiolide (5), linoleic acid (6), and 15-sandaracopimaradiene-7α, 18-dio1 (7). Compounds 1, 2, and 3 are acridone alkaloids; 4, 5, and 6 are sesquiterpenes, while 7 is a diterpenediol [5, 37, 85]. In Asia and America, several studies have reported pure compounds isolated from medicinal plants to have promising antimycobacterial activity [78, 115–117]. For example, Bisbenzylisoquinoline alkaloids from *Tiliacora triandra* (tiliacorinine, tiliacorine and 2′-nortiliacorinine) were found to have comparable antimycobacterial activity (MIC = 0.7–6.2 μg/ml) to the standard first line drugs against sensitive and resistant Mtb strains [108]. Rukachaisirikul et al. [118] reported that 5- hydroxysophoranone (an isoflavone from *Erythrina stricta*) had promising antimycobacterial activity (MIC = 12.5 μg/ml) against Mtb H_37_Ra. Vasicine acetate and 2-acetyl benzylamine isolated from hexane extract of *Adhatoda vasica* Ness. (Acanthaceae) inhibited one sensitive and multidrug-resistant strain at 50 and 200 μg/ml respectively [119]. Since flavonoids and alkaloids were reported to be absent in three out of the five inactive plants [28] and majority of the isolated bioactive pure compounds belong to the class of alkaloids, terpenoids, and flavonoids [5, 85, 118], it implies that these classes of phytochemicals are the ones most likely to be responsible for the observed antimycobacterial activity.

## Conclusion

East Africa has a rich diversity of medicinal plants that have been reported to be effective in the management of symptoms of TB. Most of the plants are from the family Fabaceae, Lamiaceae, and Asteraceae. A large proportion of the documented plants have not been scientifically validated for their efficacy and safety. Although the standard drugs had superior activity, majority of the validated plants were found to possess acceptable acute toxicity profile on animal cells and considerable bioactivity with isolated pure compounds showing promising efficacy against Mtb. We recommend more scientific validation studies to be conducted on the remaining plants in order to standardize herbal medicine use and also promote drug discovery and development against TB. More isolation and characterization studies will enrich the chemical diversity of both the natural product and synthetic chemical libraries from which possible lead candidates could be developed. Currently, we are working on isolation and characterization of bioactive compounds from selected medicinal plants from family Fabaceae identified from this study. These include *Erythrina abyssinica*, *Albizia coriaria*, and *Entada abyssinica*.

## Supplementary information

**Additional file 1: Figure S1**. PRISMA flow diagram used for the review.

## Data Availability

This is a review article and no raw experimental data were collected. All data generated or analyzed during this study are included in this published article.

## Abbreviations

IC_50_: Median cytotoxic concentration
LD_50_: Median lethal dose
Iso: Isoniazid
MIC: Minimum inhibitory concentration
Rif: Rifampicin
H37Rv: Pan sensitive Mtb strain
TMC331: Rifampicin-resistant Mtb strain
SI: Selectivity Index
TB: Tuberculosis
WHO: World Health Organization
