# Supplementary material for: Ethnobotany, ethnopharmacology, and phytochemistry of traditional medicinal plants used in the management of symptoms of tuberculosis in East Africa: a systematic review
Source: Trop Med Health. 2020 Aug 14;48:68. doi: 10.1186/s41182-020-00256-1 (PMC7427981; doi:10.1186/s41182-020-00256-1)
Supplement: Supplementary file 1 — Additional file 1: Figure S1. PRISMA flow diagram used for the review. [file 41182_2020_256_MOESM1_ESM.doc]

**Screening**

**Included**

**Eligibility**

**Identification**

Records identified through Scopus, Web of Science, PubMed, Science Direct, and Google Scholar (n = 84)

Additional records identified through other sources
(n = 7)

Duplicates removed
(n = 16)

Records after duplicates removed
(n = 75)

Records excluded based on titles and abstracts
(n = 25)

Full-text articles assessed for eligibility
(n = 50)

Full-text articles excluded, with reasons (n = 9)

Articles not in English (n = 4)

Review articles (n = 4)

Did not provide any data (n = 1)

Eligible full articles
(n = 41)

Full text articles retrieved from reference list check

(n = 3)

Studies included in the review
(n = 44)

**Figure S1.** PRISMA flow diagram showing the search and retrieval steps of the study*. Adopted from:* Moher D, Liberati A, Tetzlaff J, Altman DG, The PRISMA Group (2009). *P*referred *R*eporting *I*tems for *S*ystematic Reviews and *M*eta-*A*nalyses.
